# Bringing together multimodal and multilevel approaches to study the emergence of social bonds between children and improve social AI

**DOI:** 10.3389/fnrgo.2024.1290256

**Published:** 2024-05-17

**Authors:** Julie Bonnaire, Guillaume Dumas, Justine Cassell

**Affiliations:** ^1^Inria Paris Centre, Paris, France; ^2^Research Center of the CHU Sainte-Justine, Department of Psychiatry, University of Montréal, Montreal, QC, Canada; ^3^Mila–Quebec Artificial Intelligence Institute, Montreal, QC, Canada; ^4^School of Computer Science, Carnegie Mellon University, Pittsburgh, PA, United States

**Keywords:** hyperscanning, functional near infrared spectroscopy (fNIRS), child dyads, remote social interaction, rapport, naturalistic conditions, social AI, embodied conversational agent (ECA)

## Abstract

This protocol paper outlines an innovative multimodal and multilevel approach to studying the emergence and evolution of how children build social bonds with their peers, and its potential application to improving social artificial intelligence (AI). We detail a unique hyperscanning experimental framework utilizing functional near-infrared spectroscopy (fNIRS) to observe inter-brain synchrony in child dyads during collaborative tasks and social interactions. Our proposed longitudinal study spans middle childhood, aiming to capture the dynamic development of social connections and cognitive engagement in naturalistic settings. To do so we bring together four kinds of data: the multimodal conversational behaviors that dyads of children engage in, evidence of their state of interpersonal rapport, collaborative performance on educational tasks, and inter-brain synchrony. Preliminary pilot data provide foundational support for our approach, indicating promising directions for identifying neural patterns associated with productive social interactions. The planned research will explore the neural correlates of social bond formation, informing the creation of a virtual peer learning partner in the field of Social Neuroergonomics. This protocol promises significant contributions to understanding the neural basis of social connectivity in children, while also offering a blueprint for designing empathetic and effective social AI tools, particularly for educational contexts.

## 1 Introduction

Understanding how we learn to form social bonds with others, and the roles that building of social bonds plays in our life, holds significant value as it promises to unveil the intricate processes through which individuals connect, form meaningful relationships, and cultivate a sense of belonging, ultimately contributing to personal wellbeing, societal cohesion, and the overall fabric of social interaction. As well as its importance in and of itself, connections with those around us also play a significant role in task performance where, for example, collaboration is improved when friends work together (Sainsbury and Walker, 2009). However, the picture is not all rosy, increasing even further the need to understand. While a significant literature demonstrates that when students feel a connection to their peers they learn more (Clark and Dumas, 2015; Madaio et al., 2018), there is also a literature demonstrating that some students may put so much energy into building a relationship with their peers, that they neglect the schoolwork on which they are supposed to collaborate. This is particularly the case for low-performing students, who may wish to distract their peers from collaborative work in order to avoid engaging in tasks they do not enjoy or do well (Godwin et al., 2016).

We have coined the terms “productive rapport” and “unproductive rapport” to describe the benefits and disadvantages for task performance of engaging in social bond-building behavior, adapting terms introduced by Nasir et al. (2022) to describe productive and unproductive engagement in groups of students. We employ the term “rapport”, the harmony, smoothness and warmth experienced by participants in interpersonal interaction, and that is also recognized by observers of that interaction (Spencer-Oatey and Franklin, 2009), to avoid the vagueness that can be introduced by referring simply to “social interaction”. In order for young people to flourish, it is important to understand the underpinnings of social connection, and to know how to support them in building social bonds, but also how to use social bonds to improve performance. And yet, despite an extensive body of literature on the neural correlates of mentalizing and an increasing literature on the neural aspects of social interaction, the specific neural mechanisms underpinning the formation of social bonds among peers remain unclear. In addition, there is an unspoken assumption that putting time into building social bonds is always a net positive, which impacts the kinds of analyses carried out, and the conclusions drawn from these studies. In this context, we emphasize the significance of investigating the neural synchrony between two children engaged in both social and task interaction. As well as including data on their educational performance, over the course of a longitudinal study, and over the course of middle childhood. The longitudinal study allows us to better understand the time course of rapport, while the developmental study allows us to better understand whether different behaviors contribute to social bonds with age, whether the relationship of social bonds to performance changes, and whether the areas of the brain implicated in productive rapport change with age. We choose this field of inquiry, this approach, and this age group, because it is critical to better understand how children develop the ability to build productive social bonds, and what neural structures and dynamics underlie it, as we know that social interaction is critical to linguistic, cognitive, and even neural development (Ladd, 2005; Blakemore, 2010).

The results of this study will also serve to implement a virtual peer that focuses on productive rapport. To do so, a homologous second study, identical to the first, but where one child is replaced by a social AI agent, a virtual peer or VP, then uses this VP as a scientific tool to further understand the results of the child-child study, and to also to serve as the framework for the implementation of a virtual peer learning partner. Here we use fNIRS with the one real child to investigate the similarity and differences between child-child and child-VP social and task interaction. In this sense, the work described here finds a home in the field of Social Neuroergonomics (Dehais et al., 2020), where it has been suggested that studies of the neural correlates of social interaction can serve to improve performance of machines such as VPs. In turn, it is argued, the ways in which individuals interact with these machines can inform theories of social interaction, as well as to serve as a tool for human use (here in education). Specifically we answer the call by Kostrubiec et al. (2015) and Henschel et al. (2020) to turn to human neuroscience tools, including mobile neuroimaging, to explore long-term, embodied human–AI interaction *in situ*.

## 2 Background

In what follows we first look at relevant literature from a number of fields relevant to the current study. We center our literature review on real-time social interaction, a domain characterized as the “dark matter” of social neuroscience (Schilbach et al., 2013), and of artificial intelligence (Bolotta and Dumas, 2022). The literature review leads us to lay out a framework for the study of children's social interaction in ecologically valid contexts, and to a series of claims about what we will find in such study. We then propose the design of a novel hyperscanning experimental paradigm to adduce evidence for these claims, using functional near-infrared spectroscopy (fNIRS) to investigate interactions between pairs of children aged 5 to 12 communicating over videoconference. Our proposed study brings together a full range of conversational behaviors, data on interpersonal rapport, task performance, and inter-brain synchrony (IBS), over a number of weeks, across middle childhood.

The framework advanced here demands a close analysis of the phenomena examined and the natural contexts in which they occur. While the cognitive science literature often relies on tightly designed laboratory studies that may or may not transfer to real world situations, studies of conversational interaction “in the wild” may carry out fine-grained annotations of behavior (Schegloff, 2007). Here behaviors in different modalities are annotated and analyzed for their function in conversation: patterns of language (Schegloff, 1968; Schegloff and Sacks, 1973), non-verbal behaviors, such as mutual eye gaze (Goodwin, 1980, 1986), and what are called paraverbal behaviors, such as shifts in speech rate (Walker, 2017). In order to understand the role of these behaviors in bringing about particular psychological states, such as lateral head tilts that may convey empathy (Ambady and Weisbuch, 2010), social psychologists instead correlate the instances of behaviors with independent ratings of putative psychological states. Here a well-validated technique divides videos of people interacting into 30 second “thin slices”, presents them to untrained independent observers, and collects ratings for levels of particular psychological states. The idea behind this approach is that 30 second thin slices of video are too short to give the annotators time to reflect on what behaviors are leading them to a particular rating of rapport. This therefore evokes more rapid and thus more holistic ratings of psychological states that, by definition, cannot be viewed, but only inferred (Ambady et al., 2000). We have previously looked at the range of behaviors across modalities that are described by conversational analysts, but have correlated them with, and used them to predict, thin slice ratings in order to discover their function in bringing about psychological states. For example, we have identified what behaviors across modalities bring about the state of curiosity in small groups of children (Sinha et al., 2022).

While previous research in the field of social neuroscience has explored subjective self-reported rapport (Nozawa et al., 2019) and related phenomena in teacher-student interactions (Zhang M. et al., 2020), such investigations have not extended to peer dyads and have yet to incorporate an analysis of the behavioral features of the interaction. However, the behavioral correlates of rapport are abundantly studied in social psychology (Tickle-Degnen and Rosenthal, 1990, and following), and in linguistics (Spencer-Oatey, 2005, and following). Rapport has also been studied in the context of AI, where studies have demonstrated that it is possible to automatically recognize it (Madaio et al., 2017a), and to automatically generate behaviors that elicit it in conversational agents (Abulimiti et al., 2023). Meanwhile, in neuroscience a focus on social interaction continues to gain momentum.

While a lay use of the term sometimes refers to a feeling of “instant rapport”, for the most part rapport is a psychological state that develops over time. Because of the significant body of prior work, and the identification of specific behaviors that appear to play a role, and whose use continues to develop over the period of middle childhood (Baines and Howe, 2010), rapport would seem to be an ideal concept to anchor a developmental study. Indeed research shows that children in this age group are sensitive to quite similar phenomena, such as affiliation and togetherness, and employ behaviors that involve two people, such as synchrony of movement (what we call “dyadic behaviors”), to identify social bonds (Bowsher-Murray et al., 2023). They also accept or reject peers in part based on their ability to use the conversational behaviors that play a role in rapport (Black and Logan, 1995). At the same time, middle childhood is a time of structural and functional social brain development (Decety and Cowell, 2016; Rice et al., 2016). And yet, outside of pathologies, the topic of children's social competence and actual social interaction with peers is quite new in both the behavioral sciences and neuroscience (Ladd, 2005).

In prior work we have found the following to be particularly significant in the building, maintaining and destruction of rapport: the verbal behaviors called conversational strategies, such as self-disclosure and reference to shared experience, the non-verbal behaviors of eye gaze, head nods, and facial expressions such as smiles, and the paraverbal behaviors of laughter and pitch excurses (large variations in intonation). In each case, reciprocal instances of these behaviors (where both interlocutors engage in the behavior at roughly the same time—laughing together, or disclosing intimate information one after another—and conjunctions of these behaviors across communication modalities (what is called “multimodal communicative behavior”), appear to carry the most weight (see Cassell, 2000; Schegloff, 2007 for a general discussion of conversational behaviors). In such analyses it is essential to require ground truth as to the strength of the psychological state of rapport, so that the behaviors and psychological state are independently assessed. To this end, the level of rapport between two individuals relies on assessment by naive observers, given a general definition of rapport, and using the thin slice technique pioneered by Ambady and Rosenthal (1993). This is in contrast to some recent neuroscience literature where annotators are told to use particular non-verbal behaviors, such as mutual eye gaze or head nods, to rate rapport. It is important to consider that conflating the presence of non-verbal behaviors with social bonds risks circularity in the analysis process.

In the neurosciences a considerable body of literature has focused on the cognitive roots and neural signatures of social interaction in adults, demonstrating, for instance, the complementary roles of the mentalizing system (MS) and the mirror neuron system (MNS) (Sperduti et al., 2014; Sadeghi et al., 2022) including during peer-learning (Clark and Dumas, 2015). Within these systems, several key regions emerge: the prefrontal cortex (PFC), the superior temporal sulcus (STS), and the temporo-parietal junction (TPJ) (Molapour et al., 2021). The PFC plays a key role in social decision-making and emotion regulation (Franklin et al., 2017), processes which allow individuals to engage in prosocial behaviors that may strengthen social bonds. The superior temporal sulcus (STS) region is well-known for its involvement in a wide array of processes required for social interaction, such as understanding another's actions and beliefs, language, face perception, and the interpretation of others' eye gaze (Carlin and Calder, 2013; Monticelli et al., 2021). Finally, the TPJ, with its involvement in theory of mind processes (Saxe and Kanwisher, 2003), plays a role in perspective-taking, fostering the ability to engage in the kind of cooperative interactions essential for forming meaningful social connections. Moreover, the TPJ has been recently demonstrated to serve as a hub between self- and other-related behavior at the sensorimotor level, and a key input to the PFC for a more representational level (Dumas et al., 2020). However, studies of the mentalizing process focus in large part on single individuals, adducing evidence for hypothesized prerequisites for social interaction, and not social interaction itself, as a process co-constructed in real time by two or more participants. There are now, however, relevant technologies to study actual social interaction, such as hyperscanning; that is, the simultaneous measure of brain activity of at least two individuals engaged in interaction (Montague et al., 2002). And there are now relevant techniques to analyze social interaction. These include dyadic data analysis (Kenny et al., 2006), analyses that take the dyad as the unit of analysis, and the coordination dynamics framework (Tognoli et al., 2020), that allows social dynamics to be understood at multiple levels of description. In fact, researchers have increasingly argued, not to discount the importance of social perceptual processes, but to also integrate phenomena that require looking at two or more interactants at a time. This argument has recently been made for cognitive science (Dingemanse et al., 2023; Hamilton and Holler, 2023). It was first made for neuroscience in a visionary article by Hari and Kujala (2009), reiterated by Dumas et al. (2010), and Schilbach et al. (2013), and it has been echoed repeatedly since then, including in a recent special issue (Schirmer et al., 2021). In each case, the authors call for studies that involve two or more people engaging in interaction with one another in real time, and they call for analyses that look at the dyad as the unit of analysis, as well as the role of the individual in the dyad.

At the intersection of cognitive science and neuroscience lie experiments that deploy the tools of each to both better understand and better support human social interaction in all its complexity. We specifically argue here that a better understanding of the neurobiological basis of social interaction between children is necessary, including how the building of social bonds with peers develops over the course of multiple interactions, and over middle childhood. Since learning is the primary “job” of children, as a part of this investigation we also examine how certain types of rapport may increase learning while others may be detrimental. These topics require a dyadic perspective of the kind described above, as well as a perspective that implicates multiple levels of data—conversational, psychological, and neurobiological, as well as multiple approaches to understanding their conjunction. As hyperscanning research has progressed and continues to grow rapidly (for reviews, see Babiloni and Astolfi, 2014; Czeszumski et al., 2020), including through the use of fNIRS (Pinti et al., 2018, 2020; von Lühmann et al., 2021), it has become possible to evaluate theories concerning the nature of social interaction through *in-situ* experimentation by enabling the concurrent examination of multiple brains. This is achieved through the measurement of IBS, which assesses the temporal coherence and/or consistency in phase and amplitude of neural or hemodynamic signals across a specified time period (Dumas et al., 2010). The advances in electroencephalography (EEG) and fNIRS hyperscanning have allowed recent studies to more directly address the neural signature of social interaction, including a number of studies looking at conversational behaviors. Jiang et al. (2012) found that IBS was more present in the left inferior frontal cortex during face-to-face dialogue than when sitting back-to-back, suggesting a role for non-verbal behavior. More recently, mutual eye gaze has been identified as a potential modulator of IBS in several studies (*inter alia;* Dikker et al., 2017; Cañigueral and Hamilton, 2019; Piazza et al., 2020). Kinreich et al. (2017) report the first hyperscanning study integrating detailed annotation of non-verbal behaviors—what they call gaze and positive affect. They found that IBS was present in romantic couple dyads and not stranger dyads, and that gaze was positively correlated with IBS, while expressions of positive affect were weakly correlated, with no effect found for speech content. However, affects are underlying psychological states, and are themselves correlated with a number of different observable non-verbal behaviors, which may have therefore obscured a correlation. In addition, content of speech was not defined, and specific functions of talk may be more related to IBS than others, as described above. This is also suggested by Nguyen et al. (2021b) where an fNIRS hyperscanning protocol examined free discussion between pre-school children and their mothers. Turn-taking, conversational relevance and content contingency were assessed and only the number of turn-taking instances was correlated with IBS. This study is one of few that examine the temporal dynamics of IBS. Results showed that IBS increased more over time in mother-child dyads than in random pairs, and increased turn-taking throughout the 4-min conversation was associated with greater IBS later in the conversation. However, the presence of silences was used to assess turn-taking, while research has shown that non-verbal behaviors such as eye gaze also play an essential role (Kendrick et al., 2023), and that turn-taking can include significant overlap in speech, as well as silences. For an informative review of studies such as these, see Kelsen et al. (2022).

While the research described in the previous section focuses on conversation for its own sake, another line of research has examined the association between IBS and performance. Here, for instance, in looking at problem-solving tasks carried out independently and in teams of four individuals, it was found that IBS, as measured by EEG hyperscanning, predicted collective performance, even when the team members did not self-report strong cooperation (Reinero et al., 2021). Mayseless et al. (2019) also found a relationship between cooperation and IBS, such that as cooperation increased over time, IBS decreased (see also the recent meta-analysis by Czeszumski et al., 2022). These findings parallel those from the rapport literature, where research has highlighted the increasing importance of coordination over the course of a relationship (Tickle-Degnen and Rosenthal, 1990), but where increasing coordination almost paradoxically is accompanied by reduced use of coordination *behaviors* such as head nods (Cassell et al., 2007), as the dyad has perhaps less need of them to coordinate. Just as strikingly, a study of college students in a lecture and discussion neuroscience class used a novel protocol to assess group IBS over the course of an 11-session semester. Results demonstrated that the extent of IBS across students predicted both student self-reported engagement in the class and the social dynamics of the group, as measured by both individual and group characteristics (Dikker et al., 2017). However, as the authors themselves emphasize, it is difficult to disentangle engagement from joint attention, as all students were participating in the same activities. In addition, the study did not integrate a measure of student performance, and student engagement by no means predicts performance (Nasir et al., 2022). A number of other studies allying IBS and education have found significant neural synchrony between teachers and students, starting with Holper et al. (2013), who showed increased synchrony when the lesson was successful (see also Zheng et al., 2018; Sun et al., 2020). This literature parallels our own research showing that rapport, both directly and as a mediating variable for conversational behaviors, improves performance on collaborative tasks (Sinha and Cassell, 2015a; Madaio et al., 2018).

While the studies summarized above demonstrate a relationship between IBS and some aspects of social interaction and some conversational behaviors, each study is limited in the behaviors it examines and the modalities included (among verbal, non-verbal, and paraverbal), limited in the nature of the interaction between the participants, and limited in the analysis of how IBS changed over time. In addition, to the best of our knowledge, while hyperscanning experiments have looked at teacher-student dyads and caregiver-child dyads, experiments with child-child dyads have not been previously published.

## 3 Framework

The research summarized here leads us to propose a next step in understanding the neural signature of social bonds through a *multimodal, multi-level, dyadic*, and *temporally-sensitive* framework that provides quantitative testable predictions. By multimodal we mean, as it is used in the social sciences, linguistics, and artificial intelligence literature, multiple communication modalities. Here we include verbal (language), non-verbal (such as eye gaze), and paraverbal (such as intonation). We use the term multilevel to refer to the set of data types that includes conversational behaviors, psychological states, task performance, and brain activity. By dyadic we mean the analysis of reciprocal and mutual behaviors and psychological states. And by temporally-sensitive we refer to analyses that take into account the time of a single interaction, the 6 weeks of a longitudinal study, and the period of middle childhood. The predictions derived from the framework can be summarized with the following 6 claims:

- ***Claim 1: Conjunctions of conversational behaviors across modalities better*** ***predict IBS*. **Understanding how social bonds among peers are reflected in IBS requires the integration across the conversational modalities: verbal, non-verbal, and paraverbal, rather than looking at one single conversational modality or behavior (such as eye gaze).
- ***Claim 2: IBS and rapport evolve similarly across time*. **We use Dynamic Time Warping to quantify the temporal similarity between the two representations of social connectedness, through dyadic measures at the neural and psychological level.
- ***Claim 3: IBS Granger-causes reciprocal conversational behaviors*. **Beyond temporal similarity, we expect that IBS is a precondition of successful dyadic conversational behavior and thus the emergence of rapport.
- ***Claim 4: Not all rapport is productive*. **We introduce the notion of “productive rapport”; that is, rapport that leads to learning gains in educational tasks, in contrast to unproductive rapport that is detrimental to learning. We therefore run analyses that examine whether there is a inverted U-shaped curve between rapport-building and performance.
- ***Claim 5: Rapport-building is a process that takes time*. **Rapport is rarely built in a 1-h session, and so we argue that a study design must illuminate the time course of rapport at both the time scale of an hour during a single interaction, and of several weeks in a longitudinal design.
- ***Claim 6: Brain regions evolve with age*. **It is well-known that the brain evolves across the period of middle childhood and beyond (Lebel and Beaulieu, 2011). We argue that with age we will find more reciprocal conversational behaviors, as children become more able to participate in interpersonal synchrony, and increasingly able to develop abstract shared mental representations with others. We predict that this will lead to greater IBS, and a progressive shift in activation from rTPJ to PFC.

Bringing these axes together we predict that we will find stronger and more generalized IBS where conversational behaviors (verbal, non-verbal, and paraverbal) are reciprocal. That is, in cases of mutual eye gaze and mutual smiles, reciprocal self-disclosure, and entrainment (sometimes called alignment) where the pattern of behavior is increasingly similar between members of a dyad, such as increasingly similar speech rate. We predict that these patterns of dyadic behavior will be followed by increased IBS and that, in turn, this will allow us to distinguish between productive and unproductive rapport on the basis of IBS that follows dyadic behavior. That is to say, IBS will be present in more regions of interest, and will be stronger, when it co-occurs with high rapport that accompanies (and just precedes) learning gains.

Here, due to its non-invasive nature, high safety profile, reduced sensitivity to participants' motion, and higher spatial resolution than EEG, the fNIRS technique can play an important role (Providência and Margolis, 2022). Its reduced sensitivity to movement makes it ideally suited to investigate the naturalistic phenomena that make up social interaction (Pinti et al., 2020), and particularly social interaction among children, who may find it difficult to stay completely still. While EEG captures responses to individual events at a high temporal granularity (single syllables, articulation of the face), fNIRS integrates over these to reveal the different brain region that are more or less active on the timescale of seconds, and therefore allows us to focus on overall task components, rather than events at the sensory modality level of resolution.

An earlier study conducted by Rabinowitch and Knafo-Noam (2015) found that ratings of closeness were higher for children who participated in a synchronous finger tapping exercise, a result thought to illustrate the positive effects of synchronous (non-conversational) interaction in children, but given the increased focus in neuroscience on actual social interaction, and the tools available to do so, it is time to study IBS in child dyads engaging in actual social interaction, with an eye toward better understanding and better implementation of supporting technologies.

In what follows we therefore propose a novel study and concomitant methodology to address some of the questions raised by prior work, and to adduce evidence for the claims laid out above. We have adapted an ecologically valid interactive task used in our prior work (Finkelstein, 2018; Cassell, 2022), attractive to children across middle childhood, that can be carried out by one child (solo phase), and by dyads (collaborative phase), while attached to fNIRS apparatus. The task has variations that allow children to engage several times over a period of weeks, each time with a solo phase and a collaborative phase, and calling on slightly different skills each time, but demanding the same amount of effort. In a subsequent study, the same tasks can be carried out by a child-virtual peer dyad (as has been demonstrated in our prior work). Because the results will also inform the design of that VP, which will interact with its child interlocutor through a computer screen, all phases of the child-child experiment take place via videoconference. This also facilitates matching children with strangers of the same age, so that all evaluations of rapport are based on interactions between children who have never before met. A recent study of interaction across videoconference has shown that neither mimicry nor levels of trust are significantly different from face-to-face conditions (Diana et al., 2023). However, other studies suggest that remote interactions exhibit reduced levels of IBS compared to in-person interactions (Schwartz et al., 2022). This observed reduction might be associated with the reduced turn-taking found in Zoom interactions (Balters et al., 2023), and is postulated to be due to a decrease in the intensity of face processing and social interaction (Zhao et al., 2023). Nevertheless, assessing IBS in dyads communicating via videoconference remains feasible (Wikström et al., 2022) and important, given the increased presence of videoconference interactions in everyday life.

## 4 Experimental paradigm

Analyzing children's behavior in natural settings is the most ecologically valid method of assessing how they build social bonds (Elliott and Gresham, 1987). Consequently, the methodology we propose involves a combination of familiar tasks, comprising hypothesis construction and free discussion. A videoconference setup will include a camera and high-quality microphone, allowing us to collect high-fidelity video and audio during the entire course of the experiment (Figure 2A), that we can then analyze (in large part automatically). Our data analysis pipeline uses new methods for allying the multiple streams of data: verbal, non-verbal and paraverbal behaviors in conversation, levels of rapport, task performance, and IBS.

### 4.1 Target population and sample

An *a priori* power analysis was conducted using G^*^Power 3.1.9.7 (Faul et al., 2007) to determine the sample size needed for the study. The analysis was based on an ANOVA with 4 age groups and 6 measurements, using an effect size of (d = 0.24), based on our pilot study results, and an alpha of 0.05. The results indicate that a total sample size of 176 dyads will be required to achieve a power of 0.95. This aligns with the recommendation of Bizzego et al. (2022), who suggested a minimum sample size of *N* = 150 to detect significant IBS. There are significant challenges in recruiting participants for hyperscanning experiments, and particularly for recruiting dyads of unfamiliar children. In the event of a smaller sample size, we can adopt an analytical approach, such as the non-parametric bootstrap test with a pooled resampling method, as proposed by Dwivedi et al. (2017), and cited in Bizzego et al. (2022), which has demonstrated satisfactory performance in cases of small sample sizes and non-normally distributed data. We will enlist equal numbers of dyads from 4 age groups: 5–6, 7–8, 9–10, and 11–12 years (44 dyads per group). As social interaction may differ between boys and girls at these ages (Underwood et al., 1999), dyads will be of the same gender (both boys or both girls), and half of our participants will be male and half female (88 dyads of girls and 88 dyads of boys). As described above, in order to assess the initial building of rapport, all children will be strangers to one another. Our videoconference set-up will facilitate this, as we will set up video-conferencing in 2 different schools or after-school programs.

### 4.2 Task description

Adducing evidence for the claims outlined above places a number of constraints on the kind of task we can use: in order to investigate neural synchrony during collaboration, the task must allow both a solo and collaborative mode, so that we can compare IBS between the two and ensure that it is not due to looking at the same stimuli (Hasson et al., 2008). The task also needs to encourage social exchange, as well as to be engaging for children in a quite wide age-range (5–12 years old). In order to measure performance, the task must have answers–either tasks with correct answers, or where the nature of answers can be assessed. The age of the participants indicates that they will have a relatively short attention span, meaning that the task must be able to be completed relatively rapidly. On the other hand, since a relatively rapid task may not give enough time for rapport to develop, we have designed a longitudinal component, whereby participants engage in slightly different but comparable tasks, spending 2 weeks on each of three tasks, for a total of 6 weeks, as we have done in prior research (Finkelstein, 2018; Madaio et al., 2018; Cassell, 2022). In order to collect high-fidelity data, the task needs to be displayed on the external screen in front of the child (rather than on a piece of cardboard laid on a table, for example) so that the child's face and body are oriented toward a webcam and microphone placed next to the monitor (the computer and keyboard will be located elsewhere to diminish distractions). Finally, because a subsequent study will integrate an AI learning companion that is based on optimal performance in the child-child task, the task must allow a virtual peer agent to collaborate with the child, as well as allowing two real children to participate.

Keeping all of these constraints in mind, we therefore designed a relatively simple paradigm: a set of three tasks (described below) that ask children to generate hypotheses and evidence for their hypotheses. The children work on each of the three tasks for 2 weeks, meaning that the entire study takes 6 weeks.

Specifically, in the first, solo, phase the children will be asked to describe out loud as much evidence as they can muster for claims they are making. While this kind of task may sound too scientific for 5–6 year old children, a significant body of literature describes children's ability to do just this, albeit without necessarily using the words “hypothesis” or “evidence” (Sodian et al., 1991; Ruffman et al., 1993). Indeed, requesting scientific thinking of this sort is a common style of educational activity for the entire age group. If the participants are still generating answers at 8 min they will be encouraged to wrap up, and after 10 min they will be stopped.

Next the children will be invited to collaborate by sharing their ideas with the other child in the dyad, in order to come up with a final list of claims and evidence for those claims that they will later present to an experimenter.

After the collaboration phase, the children will engage in a social phase, where they will be asked to chat with one another in free discussion for 8–10 min while the experimenters “get their paperwork together”.

**Figure 1:**
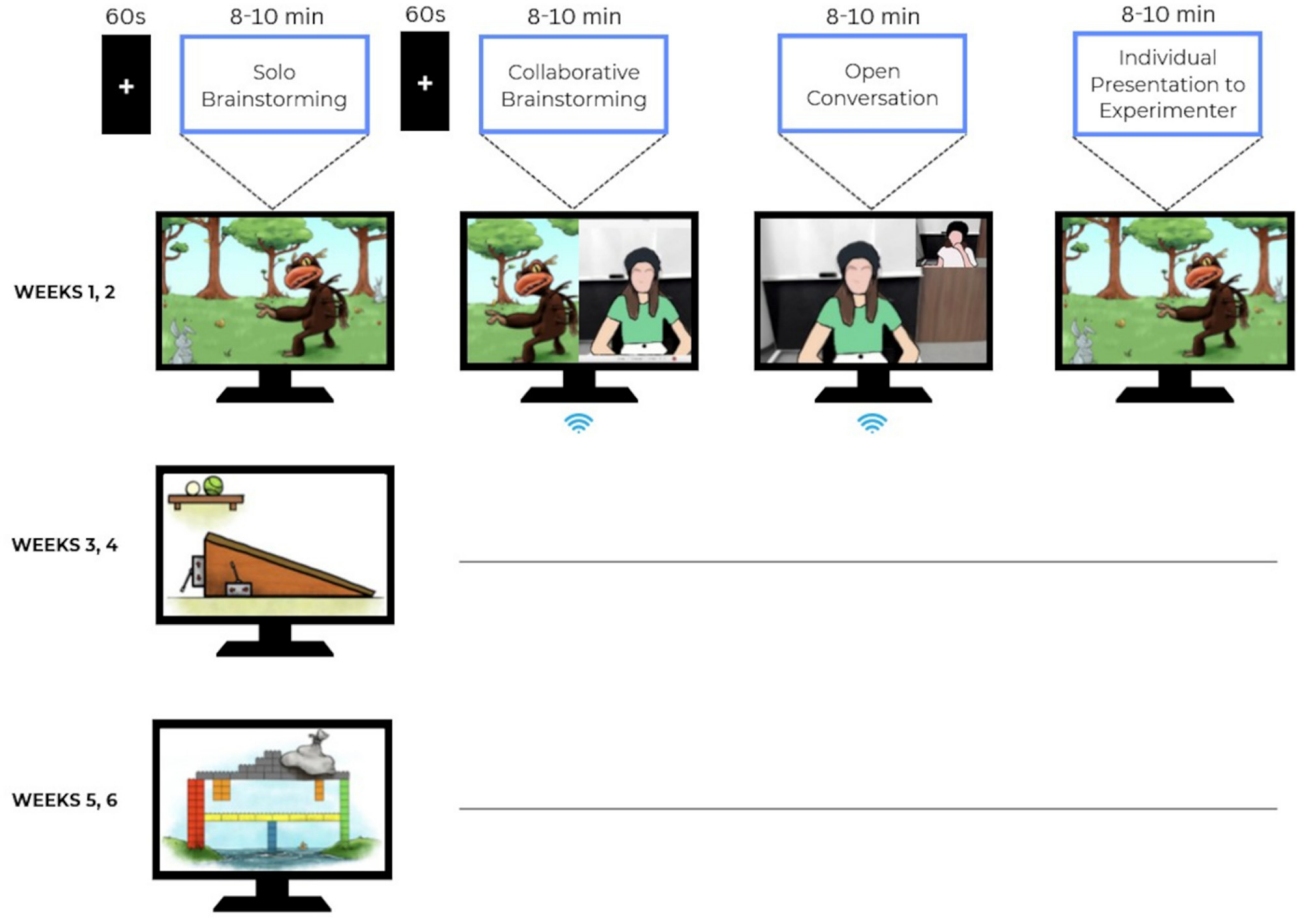
Experimental paradigm of proposed study.

After the social phase, we will turn off the children's cameras and ask them to self-report their feelings of closeness to the other child in two ways: first by rating their level of preferred closeness to the other child in the dyad, using a well-validated preferred closeness scale for children (Strayer and Roberts, 1997), and then by completing their feelings of closeness using the “Inclusion of Other in Self” (IOS) scale (Aron et al., 1992; adapted for children by Rabinowitch and Knafo-Noam, 2015). They will then present their task results to an experimenter located locally in each of the two venues, for no more than 8–10 min. The experimenter will give no feedback other than continuation markers such as “uh huh”. This phase will serve to assess the children's learning gains after each week. Figure 1 summarizes the experimental paradigm.

The first task, used during weeks 1 and 2, will present children with an image of an imaginary animal in a pastoral environment, and will ask them to make inferences about the creature's survival habits with regard to food, protection, and movement based on its appearance and the environment depicted. The second task, presented during weeks 3 and 4, depicts a girder and beam bridge made from square blocks. The abutments and piers are of unequal widths and are spaced unequally. Children will be asked about structural changes that would allow the bridge to support more weight. The third task, presented during weeks 5 and 6, depicts a ramp with a tennis or golf ball that could roll down the ramp. The length, degree of incline and material used on the surface of the ramp are shown to be adjustable. The children will then be asked to determine how the ramp parameters could be set to maximize the speed of a ball rolling down the ramp. The tasks are depicted in Figure 1.

In week one, prior to the solo phase, children will undergo training, in which the rules and mechanics of the tasks are explained and they will be guided through describing claims (what they think) and evidence (why they think that). The training is neither recorded nor timed and children are encouraged to ask any questions that they have about the experiment. After the training, during which fNIRS capping will also be carried out, fNIRS recording will begin and a first baseline recorded, during which the children will be asked to look at a white crosshair on a black screen for 60 seconds to determine resting brain activity. A second baseline will be collected before the collaborative phase of the game, and the mean of the two baselines will serve as the resting brain activity of each child.

While 176 dyads will engage in the phases described above, a small number of dyads will serve as a control. These children will engage only in solo work, and in a report to the teacher each week, with a pre-test and post-test as above, in order to differentiate between the impact on learning gains of simply engaging in the task and that of collaborating on that task. These tasks, annotation schemes and performance metrics, have been successfully used in both a child-child and child-VP longitudinal study with 7- to 8-year-olds (Finkelstein, 2018; Cassell, 2022), and have been successfully pilot-tested with 5–6 year-olds.

Because of the complexity of recruitment for longitudinal studies, and particularly those using neuroscientific methods with children, we will start by collecting data with 9–10-year-old dyads, and expect to analyze and publish the results from that work while collecting data from other age groups.

## 5 Data collection

### 5.1 fNIRS data acquisition

**Figure 2:**
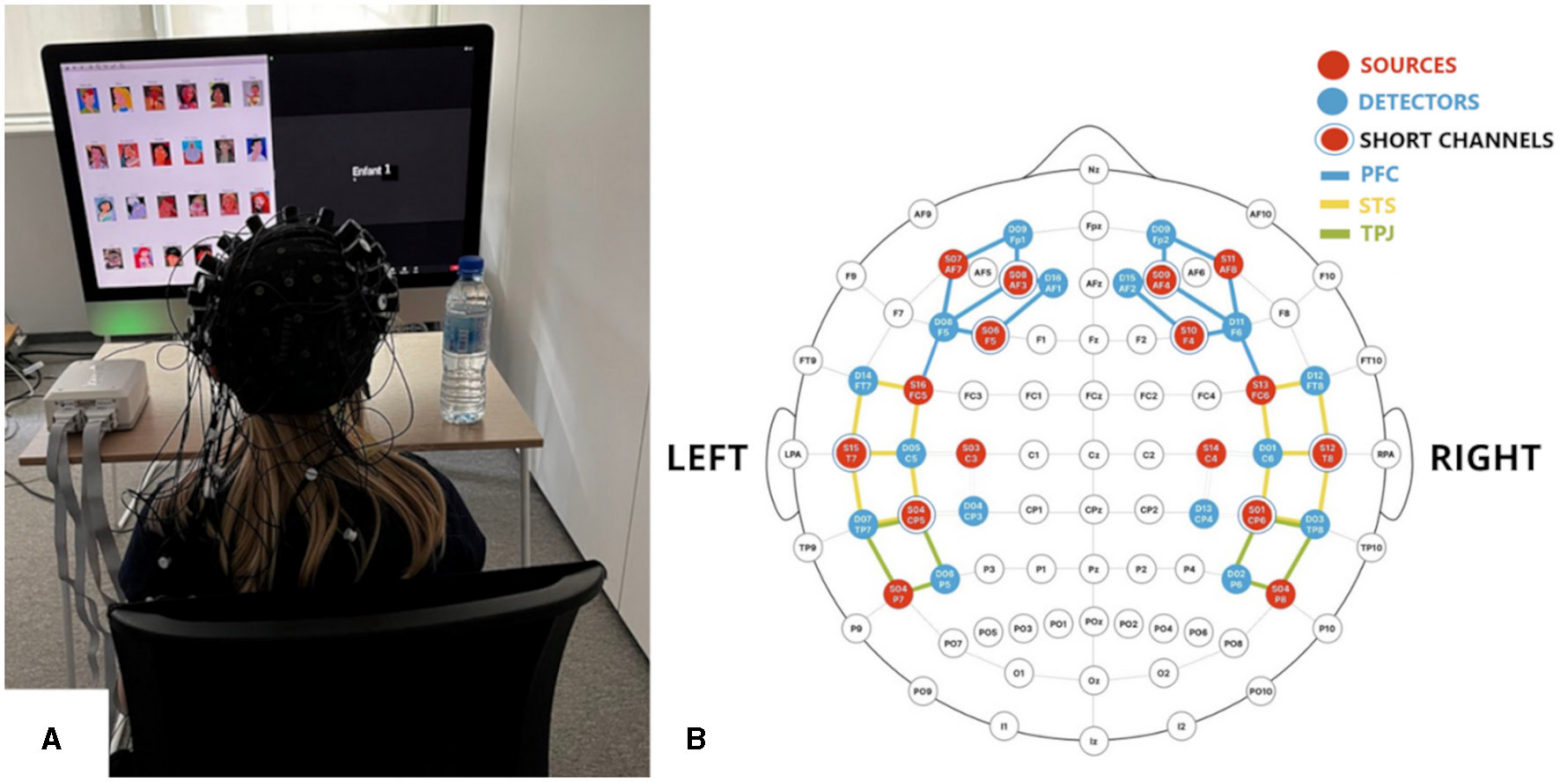
**(A)** Representative set-up of one of the experimental rooms (here in the feasibility study). **(B)** fNIRS montage showing the regions of interest: PFC (blue channels), STS (yellow channels), and TPJ (green channels). Red and blue circles represent the sources and detectors, respectively.

Functional NIRS data will be collected using two NIRx NIRSport2 machines. Each child will be connected to a separate machine with 16 sources and 16 detectors, using a sampling rate of 7.81 Hz, and wavelengths of 760 nm and 850 nm. The imaging montage of the fNIRS caps (pictured in Figure 2B) covers the 3 areas implicated in social interaction described in prior work above: the left and right PFC, STS, and TPJ. Eight short channels (8 mm source-detector separation, illustrated in Figure 2B) will be used to address systemic physiological artifacts (Wyser et al., 2020). Neural signals will be recorded using the NIRx Aurora and Hyperscan software packages, which automatically synchronize the recording from both NIRSport 2 machines via an *ad hoc* network. Because children may vary in head size, even within one age group, optodes that are destined to be placed in the same ROI may in fact capture data from a different region. Improved precision in channel localization will therefore be addressed by pinpointing the locations of the channels for each child in terms of the MNI coordinates, through digitization of fNIRS optode locations, as detailed by Hu et al. (2020). This method facilitates accurate spatial mapping of neural activation across participants.

### 5.2 fNIRS data pre-processing

For each subject, raw data will be visually inspected for evidence of artifacts that would result in poor data quality. Pre-processing will be carried out by MNE-NIRS, a dedicated Python library for pre-processing fNIRS data (Luke et al., 2021). To further enhance data quality, for each source-detector pair, the impact of systemic physiological factors will be attenuated by performing short-channel regression using the temporally embedded Canonical Correlation Analysis (von Lühmann et al., 2020). Raw intensity values will be converted into optical density values and then motion-corrected using temporal derivative distribution repair (Fishburn et al., 2019). The scalp coupling index (SCI), a measure of signal strength between the optodes and the scalp (Pollonini et al., 2014), will then be calculated for each of the 42 channels for the entire experiment. Any channel with an SCI value lower than 0.8 will be excluded from subsequent analysis (Pollonini et al., 2016). For the subsequent dyadic neural synchrony calculations (described further below), only shared channels with sufficient SCI will be used. Remaining data will then be converted into hemoglobin concentration values using the modified Beer-Lambert law (Baker et al., 2014). Data will be filtered in order to remove heart rate and respiration artifacts by applying a band-pass filter between 0.01 and 0.5 Hz (Yücel et al., 2021). Both oxygenated hemoglobin (HbO) and deoxygenated hemoglobin (HbR) will be analyzed, as HbO exhibits greater sensitivity to changes in cerebral blood flow (Jiang et al., 2012), while NIRS data acquired during verbal communication is most accurately represented by the HbR signal (Zhang et al., 2017), since speaking can influence end-tidal CO2 blood levels (Scholkmann et al., 2013).

#### 5.2.1 Epochs

Before calculating IBS, we will segment the neural data from each phase into 30-second epochs, as described in Nguyen et al. (2021b). These 30-second epochs allow us to observe the change in IBS over time. As well as its use in prior neuroscience studies, 30 seconds is the time period used in our prior work estimating rapport, facilitating the behavior analysis pipeline we describe below.

#### 5.2.2 Inter-brain synchrony

Inter-brain synchrony will be calculated using wavelet transform coherence (WTC), which allows comparison of similarity between signals in terms of spectral content (Czeszumski et al., 2020). WTC calculations will be conducted using the hyperscanning Python pipeline (HyPyP) developed by Ayrolles et al. (2021), across six regions of interest (left and right PFC, left and right TPJ, left, and right STS) within the low-frequency range 0.01 to 0.15 Hz. This range is chosen to filter out respiration and heart rate while still allowing a relatively wide range of frequencies implicated in free verbal interaction in prior work (specifically Mayseless et al., 2019; Nguyen et al., 2021b). Like these previous authors, however, we will carry out visual inspection and spectral analyses and add higher frequencies if motivated by the data.

For each dyad, we will obtain IBS values for each 30 second epoch and each ROI pair. We will also compute the average IBS across each 8–10 min phase of the experiment. In total, we will thereby obtain IBS values for each dyad, from each ROI, for the entire phase and for each 30-second epoch within that phase. To best capture the IBS evoked by social interaction, we normalize the values of the social phase by computing a Z-score, i.e., removing the average value of the solo phase and dividing by the standard deviation. This process is repeated for each dyad. To compare the difference between phases, then, we run a general linear model (GLM). Normalized IBS values are entered as the dependent variable with interactive phases (i.e., collaboration and discussion) and the interaction effect of ROIs (six per dyad) as fixed factors. To assess whether the observed neural activity is most likely due to exchanges between the children, as opposed to being primarily driven by the content of the task, we will also generate random pairings by calculating IBS with false dyads (the child's time-series paired to the time-series of another child coming from a different dyad during the same phase). We will create multiple such random pairs to generate the distribution of IBS expected under the null hypothesis (H0) (Nguyen et al., 2021a). The values of the actual real pairs will then be compared to these H0 distributions, thus providing an empirical *p*-value. An example of this analysis is illustrated for the feasibility study in Figure 5B. We will also generate a more conservative intra-pair comparison based on temporally shuffled behavior. In this case, the participants stay the same but the epochs are randomized to generate false dyads (Ayrolles et al., 2021).

### 5.3 Behavioral annotation

We argued above that it is not sufficient to focus on one single conversational behavior, nor one single modality of conversational behaviors, in order to understand the ways in which the behaviors of social interaction are related to IBS. Here we describe how we acquire the multimodal data. Conversation is influenced by the situational context (e.g., classroom vs. playground, brainstorming vs. class presentation), and by the interlocutor (e.g., adult vs. child, stranger vs. friend or family member). These differences underscore the importance of rigorous annotation, including using automatic extraction based on advances in signal processing to allow efficient treatment of large amounts of data, as well as hand annotation using coding manuals tested and normed for the culture and age group in question.

In terms of the automatic extraction of nonverbal behavior, we will employ OpenFace, a widely-used open-source computer vision framework that utilizes action units (AUs) to detect facial landmarks, allowing us to detect facial expressions in video (Baltrusaitis et al., 2018). AUs are translated into behavioral features such as smiles. This allow us, for example, to differentiate between the Duchenne or genuine smile and the polite smile (Ekman et al., 1990). We will next perform a manual check on 10% of the data at each age using the ELAN video annotation tool (Wittenburg et al., 2006) to ensure that the behaviors are correctly annotated by the software. We have conducted such checks in the past, and have found alignment between high inter-rater reliability hand annotation and OpenFace. If, however, this is not the case for these data for any reason, we will fine-tune the software with the annotated data to ensure alignment and then retest on a second sample of 10%. Second order behaviors (e.g., mutual smiles) will then be automatically extracted. Assessment of eye gaze in the videoconference-mediated conversations will be achieved using the framework developed by Tran et al. (2022) applied to the raw eye gaze data generated by OpenFace. This application enables the extraction of eye gaze direction employing a dynamic clustering algorithm. As with facial expression, we will then automatically extract the second-order behavior of mutual eye gaze.

In addition to non-verbal behavior, we will also extract prosodic features (e.g., pitch, loudness and speaking rate), which have also been shown to play a role in rapport management (Grimberg et al., 2022). These features will be extracted using a widely used open source audio analysis framework called OpenSmile (Eyben et al., 2010), which enables acoustic features in the audio to be extracted and then interpreted as prosodic features such as intonation, speech rate, and loudness. We will then automatically extract the second order behaviors (e.g., identical speech rate, entrainment of loudness, etc.).

Finally, we will annotate verbal or linguistic behaviors using the Elan software package, version 6.7 (Wittenburg et al., 2006), concentrating specifically on what are called conversational strategies—ways of speaking that serve particular interpersonal functions (such as using praise to “soften up” one's interlocutor, or hedges to avoid embarrassing the other person), that have been shown to play a role in building rapport among adults and young people (Zhao et al., 2014; Madaio et al., 2018). Our coding scheme for conversational strategies, refined and published in numerous papers over the last decade, is based on behavioral phenomena that have been shown in work by others and by our own team to impact rapport. Each coding scheme treats one verbal behavior. In the current work, we will annotate self-disclosure (revealing details about oneself that are not publicly available, such as “I love dogs”), reference to shared-experience (referring to an event that was engaged in together, such as “last week's problem was really hard, right?”), praise (such as “you're better at this than me”), and backchannels (sounds or words that indicate that the listener is following, such as “uh huh” or “yeah”). We will then use a grounded theory (Glaser and Strauss, 1967) bottom-up approach on a subset of the data from each age to understand if we have missed any relevant conversational strategies in the dataset and we will add them to the annotation scheme if so. Following this, we will automatically extract second-order conversational strategies, such as mutual self-disclosure (“I love dogs.” “Me too”). Annotation of conversational strategies will be carried out by experienced annotators whose inter-rater reliability on Krippendorf's alpha must be above 0.7 before they proceed to annotating independently (Krippendorff, 2011). Importantly, we will annotate only what is visible, and then correlate with the underlying dyadic psychological state of rapport, rather than placing annotation of putative underlying states such as beliefs (e.g., this child likes her partner), feelings (she looks happy), and/or intentions (she wants to convince the other child that she's right) on the same level as visible behaviors.

### 5.4 Rapport estimation

We argue that analyzing only conversational behaviors with respect to IBS without associating them to underlying psychological states obscures how those psychological states are produced dyadically through particular multimodal conversational behaviors. For this reason we will carry out careful objective estimation of the level of rapport between the children in each dyad by using the “thin-slice” method, described above, in which the video is divided into 30-second “thin slice” segments (Ambady and Rosenthal, 1993; Ambady et al., 2000). These segments will be presented in random order to four independent raters on an online micro-work platform (such as Amazon Mechanical Turk or Prolific). The annotators will be provided with a simple one sentence definition of rapport as harmony and ease of interaction, and asked to evaluate each slice of video on a Likert scale ranging from 1 to 7, where higher scores indicate higher rapport (Sinha and Cassell, 2015a). A single rapport value for each slice will be produced by throwing out the rater most distant from the other three, and then calculating intraclass correlation and Cronbach's alpha among the remaining raters to determine consistency and reliability, respectively (Ambady and Rosenthal, 1993; Madaio et al., 2018). The 30-second slices will then be reassembled in their original order, giving a picture of the temporal dynamics of rapport over the course of the interaction.

While we sometimes refer to feeling instantly “in sync” with somebody we have just met, for the most part rapport fundamentally has to do with the change of a relationship over time. In order to assess the changes in rapport over the course of the 6 weeks of the longitudinal experiment, we will rely on the concept of utopy (Sinha, 2016). Prior work has shown that statistical summaries such as a measure of central tendency or proportion of high and low ratings of rapport, collapse the temporal dimension and are not as robust as more stochastic-based models which capture the evolution of rapport over time (Sinha, 2016). We will thus fit a Markov chain of order 1 to the sequence of 16 to 20 rapport ratings for each session (8–10 min), the sequence of 32 to 40 rapport ratings over the two dyadic sessions (collaboration and open-ended chat) and the 192 to 240 rapport ratings for the two dyadic sessions over the entire 6 weeks, and use the resulting transition probability matrix to generate a measure of the “utopy,” or likelihood of the dyad being in a high-rapport state. This likelihood is calculated as the sum of each transition probability weighted by the distance of the transition (Sinha, 2016; Madaio et al., 2017b). This allows us to assess the temporal dynamics of rapport.

As described above, we will also ask children after each interaction to rate their preferred closeness and feelings of closeness to their partner.

### 5.5 Educational performance

We argue for two types of rapport to be distinguished: productive and unproductive. Both types are associated with important health and wellbeing benefits, such as feelings of social cohesion. However, we define productive rapport as that which serves not only to improve wellbeing, but also to increase learning gains when two children collaborate. Unproductive rapport, on the other hand, may serve to distract the learners and reduce learning gains. To assess what behaviors identify productive rapport, we will assess the learning gains of the individuals in each dyad, and the mean learning gain of the dyad, each week and over the period of the 6 weeks, as follows: as described above, the children will individually report their hypotheses and evidence to a locally-located experimenter each week, after the collaborative and free discussion phases are finished. This “reporting out” serves as a metric of performance on each week's work. In addition, the delta between a pre-test (before the 6 weeks begin) and post-test (after the end of the 6^th^ week) will measure learning gains over the course of the 6 weeks for each individual and for the dyad in order to assess the relationship between IBS, use of conversational behaviors, level of rapport, and learning gains. The pre-test and post-test will be similar to the first task used in week 1 and 2, and they will be administered by the experimenter located in each venue. Here the experimenter will show the child an image of a new imaginary animal (a different animal for the pre-test than for the post-test), and several pictures of environments, and ask the child whether each of the environments would be a good or bad place for the animal to live in and why. After the child has given arguments for why each of the environments would be suitable or unsuitable for the imaginary creature, the experimenter will ask which of the environments would be best and why. Once again this will last no more than 8–10 min. The students' responses on the weekly read-out and the pre- and post-test will be annotated for instances of every-day science reasoning language (hypotheses, claims and evidence, expressed in lay language, such as “I *think that* he can climb trees *because* he has long claws”). The annotation will be carried out using a scoring manual for everyday science language that we developed for prior work (Finkelstein et al., 2013; Finkelstein, 2018), and that has achieved high inter-rater reliability. The manual was developed based on a significant body of prior work (*inter alia*, Lemke, 1990; Kafai and Ching, 2001; Kurth et al., 2002; Nemirovsky et al., 2004). The categories include “testing or evaluating ideas”, “causation”, “explanation”, sorting/classifying”, and “comparing”, among others. The children's interactions with one another, both during the collaboration phase and the social phase are also annotated for conversational behaviors and for rapport, as described below.

## 6 Data analysis

Intrinsic to the novel methodology we are proposing is the alignment of neural data with multimodal conversational data on the one hand, and ratings of interpersonal rapport on the other. Synchronization of the video, audio and fNIRS equipment will be carried out by event timings from PsychoPy (Peirce et al., 2019), as described above. For the analyses below, therefore, while potentially challenging due to the different time-scales of the different modalities, we use the mean calculated on each 30-second window for each data type, and can therefore synchronize the different behavior streams (audio, video, rapport estimations, and IBS) using the event codes recorded in the fNIRS data, prior to subsequent cross-data type analysis.

### 6.1 Claim 1: conjunctions of conversational behaviors across modalities better predict IBS

We argue that focusing on a single conversational behavior (such as eye gaze) under-estimates the complex interplay among modalities in their relationship to neural synchrony. To adduce evidence for this claim we will leverage Long Short-Term Memory (LSTM) networks to predict IBS on the basis of temporal sequences of behavioral data (see Table 1 for further details on the behavior types and variables). Since conversational behaviors are likely to lead to IBS with a certain lapse of time, we insert a 1 to 10 second time lag between the sequences of conversational behaviors and the IBS (Chang et al., 2022). The dataset will be structured into sequential behavioral data over time, each associated with a 30-second interval IBS value and normalized through a Min-Max scaling technique, ensuring consistent proportions across all features, while preserving the original distribution of the data.

**Table 1:** Data sheet.

**Level of data type**	**Modality**	**Dyadic status**	**Specific variables**	**Notes**
Conversational Behavior	Verbal	Non-reciprocal	◾Self-disclosure	Number of behaviors per 30 second epoch.
			◾Reference to shared experience	
			◾Praise	
			◾Teasing	
		Reciprocal	◾Mutual self-disclosure	
			◾Mutual reference to shared experience	
			◾Mutual teasing	
	Paraverbal	Non-reciprocal	◾Pitch shift	
			◾Shift in speed of speaking	
			◾Shift in loudness	
		Reciprocal	◾Mutual pitch shift	
			◾Mutual shift in speech rate	
			◾Mutual shift in loudness	
	Nonverbal	Non-reciprocal	◾Smile	
			◾Head nod	
			◾Gaze at other person	
		Reciprocal	◾Mutual smile	
			◾Mutual head nod	
			◾Mutual eye gaze	
Rapport State		Rapport rating (1–7) for each 30 second interval for each dyad		
Educational Performance		Every week	Task 1–3, week (with x = 1–6)	◾Number of hypotheses per weekly 8–10 min read-out
				◾Number of pieces of evidence per 8–10 min weekly read-out
IBS				◾Right hemisphere ROIs

The study will employ a hierarchical LSTM model to process these multimodal inputs. Within this framework, different LSTM modules will be dedicated to distinct input modalities, with their outputs subsequently merged in the following stage. Given that each modality may generate inputs at varying frequencies, we will synchronize these signals, setting a uniform frequency for the temporal signals that aligns as a common multiple of their various frequencies. To overcome the inherent limitation of traditional LSTM models, which often lose track of initial inputs over time, we will incorporate attention mechanisms known for their effectiveness in preserving memory. Additionally, the LSTM model will feature a feed-forward network capped with a softmax function at its output, aiming to categorize the hidden representations into specific conversational behaviors. The model's weight functions will be honed through supervised learning, utilizing Back Propagation Through Time (Mozer, 1995). For the training of our LSTM model, we will employ cross-entropy loss as a key metric.

Training will involve temporal cross-validation, preserving the chronological order of sequences. Post-training, we will employ two techniques for feature importance analysis: an ablation analysis to selectively remove features in such a way as to evaluate their impact on model performance, and SHAP (SHapley Additive exPlanations), a model agnostic method proven effective in our prior work (Grimberg et al., 2022), for quantifying each feature's contribution to rapport predictions. The model's performance will be evaluated on a distinct test set, with adjustments made based on results. This comprehensive approach will allow us to not only predict IBS from behavioral data but also determine the contributions of specific behavioral features and combinations of features, shedding light on the factors influencing IBS dynamics.

### 6.2 Claim 2: IBS and rapport evolve similarly across time

After describing how we will assess the relationship between reciprocal conversational behaviors and IBS, we now turn to the relationship between the psychological state of rapport, as brought about by conversational behaviors, and IBS. To achieve this, we will establish a behavior analysis pipeline that preserves the temporality of the different data streams, while highlighting the relationship among them. Because the time scales of different behavior streams may differ, it can be challenging to analyze the potentially non-linear relationship between rapport and neural synchrony. Here we will therefore set up a pipeline using Dynamic Time Warping (DTW), a powerful method that has been previously used to compare the dynamics of brain activation between the time series of two participants (Azhari et al., 2019). In our own prior work, DTW has also been used to characterize how the conversational strategy usage for each partner in a dyad is aligned in time with that of the other partner (Sinha and Cassell, 2015b), allowing us to assess entrainment, and influence of each member of the dyad on the other. The application of non-linear transformations as carried out by DTW is particularly advantageous for the relatively slow fNIRS time scale compared to the observed behavioral events (Quaresima and Ferrari, 2019). The DTW method causes specific changes in the timing of events in two different data streams. These alterations are made in such a way as to allow for the best possible alignment or matching of the two time-series (Berndt and Clifford, 1994). DTW then provides a measure called “warping distance”, which helps us compare and quantify how different the two sequences are from one another, based on the changes needed to align them. Smaller warping distance values mean that the sequences are more similar, while larger values indicate greater dissimilarity.

To assess the relationship between rapport and IBS, we will therefore calculate the warping distance between IBS and rapport values for each dyad across 30-second intervals. In order to assess the statistical significance of the observed similarity between IBS and rapport curves post-application of DTW, a permutation test will be employed. This involves randomizing the time slices of one of the curves and recalculating the warping distance, repeating this process through 1,000 iterations. The resulting distribution of permuted distances facilitates the determination of a *p*-value, which, when the significance level is set, elucidates whether the observed pattern similarity is statistically significant. Subsequently, a two-way (2 × 7) repeated measures ANOVA can be conducted to explore the effect of experimental phases (collaboration and discussion) and brain regions (left and right PFC, left and right TPJ, left TPJ, left, and right STS) on the resultant warping values. This analysis will enable the assessment of overall differences in warping values attributable to each factor, as well as potential interaction effects between experimental phases and brain regions, providing a clearer understanding of the factors shaping the relationship between rapport and IBS. After having analyzed the relationship between the temporal dynamics of rapport and IBS, we will also run a mediation analysis, where we examine rapport as a mediating factor between reciprocal conversational factors and IBS, to test how conversational factors may influence the dyadic dynamics at both neural and psychological levels. This was the case in prior research where we found that rapport played a mediating role between conversational behaviors and learning gains (Sinha and Cassell, 2015b).

### 6.3 Claim 3: IBS Granger-causes reciprocal conversational behaviors

While the previous analyses give a sense of the relationship among time courses, they do not illuminate the direction of influence between one time course and another. To that end, we will compute Granger causality (Granger, 1969). Our prior work already shows that rapport Granger-causes increasing entrainment in some conversational behaviors, and particularly in speech rate (Sinha and Cassell, 2015b). It has also been employed to show that dual brain stimulation improves collaborative learning via the mechanism of spontaneous movement synchrony (Pan et al., 2021). Here we seek to understand whether rapport Granger-causes IBS or vice-versa and, subsequently, whether conversational behaviors Granger-cause IBS or vice-versa. Given the slower time scale of fNIRS signals, a relatively large time window will be used. That is, we hypothesize that it might take as long as 5 seconds for one of these phenomena to Granger-cause the other. The Granger causality approach relies on the accuracy of one time series in predicting the future behavior of another time series. In particular, we determine whether time series *A* (e.g., IBS) Granger-causes time series *B* (e.g., rapport or a relevant behavior feature) by assessing whether incorporating past observations of time series *A* in a linear regression model of time series *B* and time series *A* reduces prediction error compared to a model containing only previous observations of time series *B*.

In recent neuroscience studies, Granger causality has demonstrated the ways in which men in elderly couples Granger-cause competitiveness in their spouses in the first half of a competitive game, while there is no causality in either direction in the latter half of the game (Zhang et al., 2023). In recently-published research, it has been shown that mutual non-verbal behaviors, particularly mutual smiles, laughter, and body movement anticipate and Granger-cause IBS, even in the absence of a specific task (Koul et al., 2023). However, in that experiment participants were *required* to look at one another, and so spontaneous mutual gaze behaviors were not evaluated. In the current work, we compute the pairwise conditional G-causality in both directions. If no causality is evident in either direction, our prior work motivates looking at the causal relationships between the mutual behaviors previously revealed to be influential in rapport-building and IBS (described above). Therefore, in this case, time series *A* and time series *B* will refer to the average IBS value for every 30-second epoch and the rapport values/occurrence of a relevant behavior for each 30-second slice. The statistical significance will then be assessed through an F-test under the null hypothesis that one time series does not Granger-cause the other. This analysis can reveal both unidirectional influences, wherein one time series significantly Granger-causes the other (i.e., one time series tends to lead the other), and bidirectional influence, indicating that both time series Granger-cause one another in a reciprocal manner. Indeed, if past values of the time series *A* help improve the prediction of time series *B*, and at the same time, past values of time series *B* also help improve the prediction of time series *A*, then bidirectional influence is present.

### 6.4 Claim 4: not all rapport is productive: adding in performance data

While rapport is often seen as beneficial in general, and in particular for collaborative tasks, its impact on task performance is not uniformly positive. To investigate this, we propose integrating performance data into our analysis of IBS, conversational behaviors, and rapport. Methodologically, we will first compute the specific performance metrics relevant to the tasks undertaken by dyads in our study, as described above. In order to associate these metrics to conversational behaviors, rapport level, and IBS, over time, we count the number of hypotheses, and evidence statements supporting those hypotheses, in the read-out session between the children and the experimenter for each week, and we associate these performance metrics to the rapport utopy score for that week, and the mean IBS score for the collaboration phase for that week. Our hypothesis is that while a moderate level of rapport is likely to be beneficial for performance, either too little or too much rapport might hinder task effectiveness. This could be due to an overemphasis on maintaining harmonious interactions at the expense of task focus, or conversely, insufficient rapport leading to poor collaboration. We therefore expect to find a non-linear relationship, potentially inverted U-shaped, between rapport levels and task performance. To test this hypothesis we will employ regression models, including quadratic regression, to test the hypothesized inverted U-shaped relationship. This will enable us to assess the impact of varying levels of rapport on performance metrics while controlling for other variables. Furthermore, we will conduct subgroup analyses to explore if this relationship varies across different dyads, tasks, levels of IBS or ages, or over the 6 weeks of the study. This could involve stratifying dyads based on one of these variables. By integrating performance data, we aim to provide a more nuanced understanding of the role of rapport in collaborative tasks. This kind of analysis may be supplemented by machine learning models such as random forests, where the model is trained on a portion of the data, with the random forests constructing multiple decision trees during the training, and outputting the mean prediction of these trees. In essence this enables the model to recognize complex patterns, such as how different variables interact to affect task performance, thus potentially uncovering intricate relationships such as the hypothesized non-linear relationship between conversational behavior, rapport, and task performance. Techniques of this sort, however, require a significant amount of data as, after training, the model is validated on a separate dataset to assess accuracy. If 176 dyads are collected, the amount of data could be sufficient. The results could have important implications for how rapport is fostered in team settings, particularly in educational and organizational contexts, where performance outcomes are critical. The insights gained from this analysis could contribute to a more comprehensive model of interpersonal dynamics, extending beyond the simple premise that more rapport is always more beneficial.

### 6.5 Claim 5: rapport-building is a process that takes time

We will employ Dynamic Network Analysis (DNA) as the simplest way to address Claim 5′s exploration of the longitudinal interplay between IBS and rapport over the course of 6 weeks. In this analysis, each dyad will be represented as a network, with nodes denoting sensors and edges indicating the strength of IBS. The temporal alignment of these networks will ensure uniformity across different time points, enabling a consistent examination of changes in connectivity. Employing dynamic network metrics, including variations in edge weights, will facilitate a comprehensive capture of evolving patterns in dyadic interactions. Visualization techniques, such as animations, will be employed to enhance the intuitive comprehension of temporal dynamics within these networks. Rigorous statistical analyses will involve comparing metrics across different time points and identifying relationships between those changes in network patterns and the evolution of rapport.

### 6.6 Claim 6: brain regions evolve with age

We hypothesize that with age we will find more reciprocal conversational behaviors, as children become more able to participate in interpersonal synchrony, and increasingly able to develop abstract shared mental representations with others, leading to greater IBS. We expect to find IBS particularly in the TPJ for the younger age groups and in the PFC in the older age groups (Dumas et al., 2020). In the absence of prior research concerning the development across middle childhood of the neural correlates of social bond formation, we propose to study IBS across the 4 age groups in relationship to the task (solo vs. collaboration vs. discussion). To that end, following Wang et al. (2022), a GLM model will be used to estimate changes in IBS values across ROIs, pairs, and distinct experimental phases. To ensure robust statistical inference, False Discovery Rate (FDR) correction will be implemented. We believe that the identification of developmental dynamics in IBS will provide valuable insights into the critical cognitive processes at play in rapport, and will thus potentially play a role in guiding the design of interventions.

## 7 Modeling productive conversational behaviors in virtual peers

With advances in end-to-end machine learning models of conversation, the promise of usable virtual reality solutions for work and play in the Metaverse, and the introduction of large language model (LLM) chatbots, there has been a renewed focus on building embodied conversational agents (ECAs), i.e., conversational agents with computer-generated animated bodies that are displayed on a screen and that can both recognize and display language and non-verbal behavior to communicate with their human interlocutors. These systems are targeting challenges as varied as adherence to medical treatment (Bickmore et al., 2010; Tudor Car et al., 2020), lifestyle changes (Kramer et al., 2020), education (Lane and Schroeder, 2022), and even daily home tasks where individuals could benefit from the support of a personal assistant (Pham et al., 2018). However, a stumbling block in the implementation of systems that can engage with their users for more than a minute or two is the implementation of the kind of appropriate rapport-building social interaction strategies that in human interaction underlie successful collaborative task performance, and that are more sophisticated than simple meaningless chit-chat. Such strategies are particularly important for educational interactions where children in the classroom and in informal learning contexts constantly intersperse task and social talk, and where, as we described above, reasonable amounts of apparently off-task social talk has been demonstrated to improve collaborative learning (Madaio et al., 2017a). The study proposed here therefore has both basic science goals, identified above, and a translational motivation, which is to build more effective Embodied Conversational Agents (ECAs) for education. The design of ECAs and specifically of virtual peers (ECAs that look and behave like children) has relied on studies of real children, including our own work on peer collaborative learning (inter alia; Madaio et al., 2017a; Cassell, 2022). The use of neuroscientific data to enhance the learning properties of ECAs has not been studied, however. In fact, the majority of studies that look at neural activation in the context of virtual agents has been restricted to people *viewing* agents and not interacting with them. Exceptions are our lab's early work comparing brain activation in individuals interacting with a human MRI technician vs. an ECA MRI technician, in two conditions: where the technician (human or ECA) simply explained the MRI, or also engaged in social interaction. In the task-only condition, increased superior temporal gyrus activation was found for the virtual human technician over the real technician. For the social interaction condition, on the other hand, interaction with both the real and virtual technicians resulted in increased activation in areas associated with social cognition, but there was greater activation for the human technician (Gayda et al., 2008). More recently, work by Chaminade et al. (2018a,b) has compared interaction with a virtual head to interaction with a person, and shown increased activation in the TPJ for the interaction with a human. However, the virtual head used in the experiment was quite limited in its expressiveness and was piloted by a “wizard” rather than being driven by human-human data. We have also found increased involvement of the rTPJ during social coordination between individuals and a virtual partner represented by the human dynamic clamp (Dumas et al., 2020).

**Figure 3:**
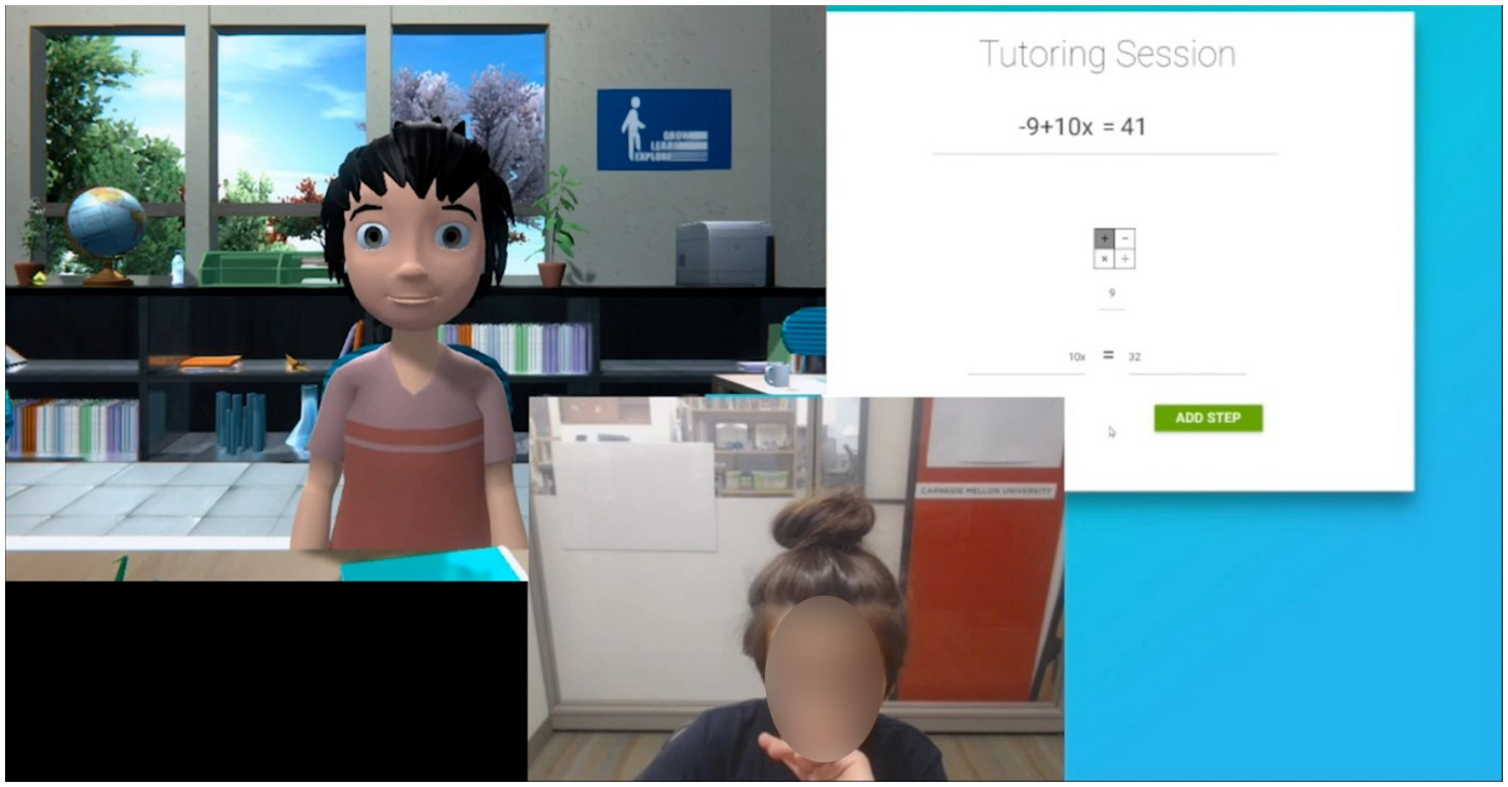
Child working with a virtual peer on a math problem (for illustration purposes).

The study outlined here is designed to better understand the relationship among conversational behaviors, rapport and IBS in pairs of children, in the context of an education-oriented task. In so doing it may thus show us the path to implementing a more effective VP, an AI system that looks (see Figure 3) and acts like a child, and that engages children in a set of conversational behaviors that can be shown to lead to productive rapport, and hence to learning gains. Implementing these behaviors in a VP gives us a way of evaluating our basic science results: do the behaviors that seem most effective in analysis turn out to be the most effective in synthesis? In the past our ECAs and VPs have always served this double role: a system that can play positive role in children's lives, and serve as a tool for better understanding the use of multimodal behaviors in interaction. In this sense, as described above, our work is tightly aligned with the goal of social neuroergonomics, that also proposes bringing together cognitive science, computer science and neuroscience in order to better understand how people engage in social interaction with one another, and how to build machines that better understand and support this interaction (Dehais et al., 2020).

As emphasized by Cross and Ramsey (2021), then, the incorporation of human-like attributes into a non-human entity, followed by the assessment of similarity using behavioral and neural measures of human interaction, offers an important way of investigating how to improve current AI systems. In our evaluation of the resultant virtual peer, we will therefore once again collect fNIRS data. Clearly, we will not collect IBS data in this context, however we will investigate the neural correlates of interaction with VPs, and compare it to that of child-child interaction for each age group.

**Figure 4:**
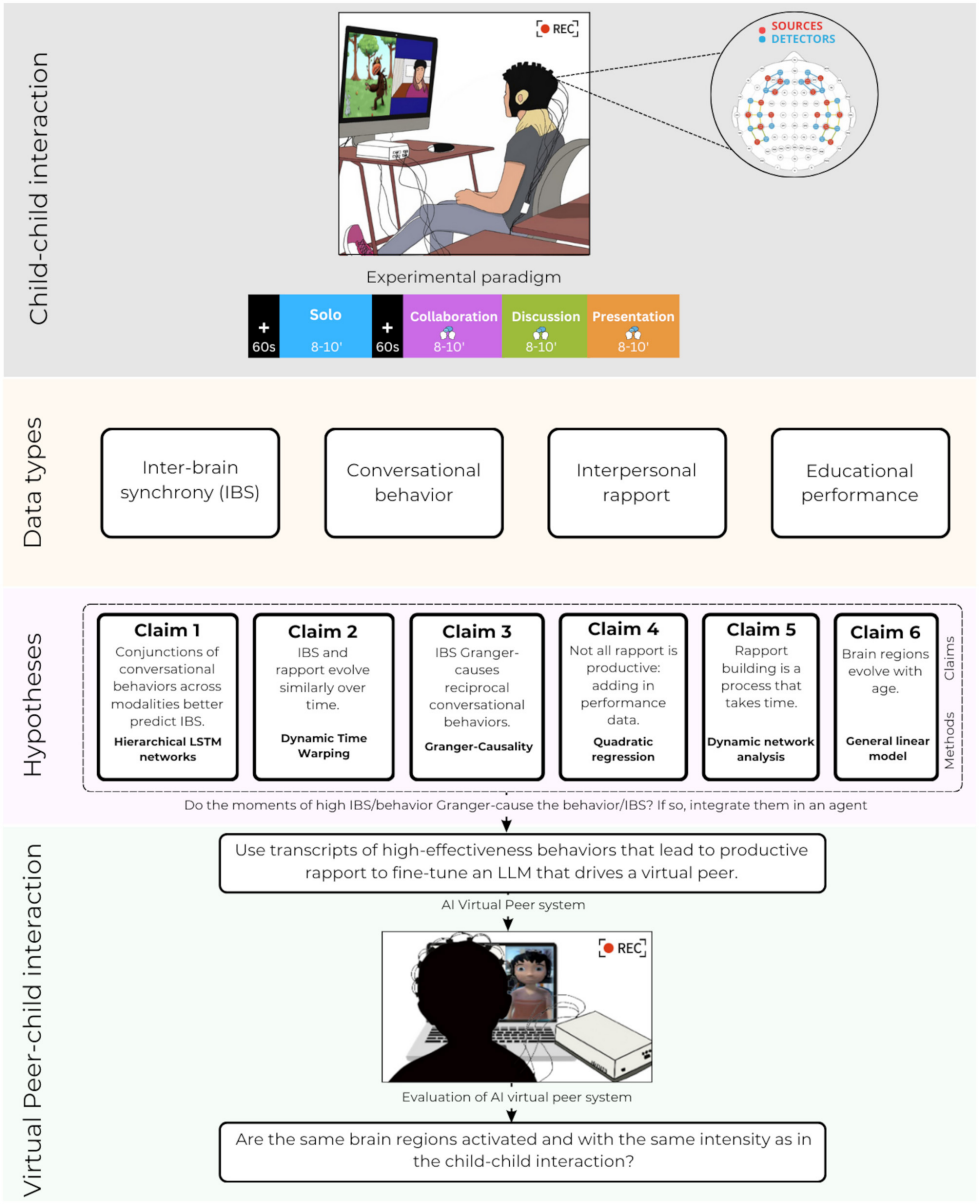
Recapitulation of the methodology.

While a full description of how to implement such VPs is beyond the scope of this article, it is important to emphasize that new techniques in artificial intelligence make this goal significantly more attainable. In particular, we have demonstrated that we can fine-tune LLMs such as DialoGPT (Zhang Y. et al., 2020) or ChatGPT3.5 (OpenAI, 2022)—that is, feed them many instances of a particular way of talking—and that the resultant machine learning model is able to then generate appropriate social language, such as hedges in the style of teenage peer tutors (Abulimiti et al., 2023). The complete methodology is depicted in Figure 4.

## 8 Feasibility study

In order to evaluate the feasibility of fNIRS hyperscanning over Zoom with dyads of children in this age group, and to assess the quality of the data collected in this manner, we ran a feasibility study. As we did not intend to look at performance metrics in the feasibility study, nor to gather data across multiple weeks, we chose to use a task that was familiar to children in this age range. We therefore developed a single-session paradigm based on a digital adaptation of the *Guess Who* game. *Guess Who* is a two-player board game that involves inferring an answer from a set of clues. The game has 24 possible characters with various distinguishing attributes (e.g., gender, facial hair, glasses, etc.). In the original board game, players randomly select a character card whose identity the player's opponent must guess by asking a series of yes or no questions. The children alternate between choosing a card, and asking questions of the other player to eliminate incorrect character possibilities on their game boards. In the experimental version, we adapted the game to be presented on a screen, with familiar characters from popular animated television and film that target this age group in France.

In addition to developing a digital version, we also adapted the game to allow both solo and collaborative modes. Thus, instead of asking the original game's yes-no questions about features pertaining to the character, children were given clues that were displayed one by one above the game board on the child's screen. In order to be suitable for pre-literate younger children as well as older, each clue was represented with a short one- or two-word label and a picture. In total, each character round consisted of four clues. Children were instructed to remove the characters that did not correspond to the clue by clicking on the character's image. In response, a red “X” appeared over the image of the character indicating that the character was eliminated. For example, if the clue was “*garçon”* (“boy”), the child saw a blue silhouette of a male human figure and should have eliminated all female characters. For the solo phase, this process was repeated for each of the four clues until only one final character was left un-eliminated. After only one final character remained on the board, the correct answer appeared in the middle of the screen. If the correct character was chosen, the character's image appeared outlined in green while if an incorrect character was chosen, the image was outlined in red. The game board then reinitialized, beginning again with a new set of clues leading to another character.

During the collaborative phase, on the other hand, each member of the dyad received different clues, which they had to compare and discuss with their partner, with whom they interacted via videoconference. For instance, one child may have received the clue “*sourire”* (“smile”) while the other child may have received “*garçon”* (“boy”). By sharing their clues, both children knew to eliminate all characters who were not male and not smiling. The game was designed to induce collaboration by making it impossible to correctly guess the final character without sharing clues.

After training and capping, the feasibility experiment was composed of four 7-min phases: solo task, first discussion, collaborative task, and second discussion. In order to allow for the capture of baseline neural activity, each experiment began with a 75-second baseline period, and there were 30-second baseline periods between each phase. Children were not in the same location and did not meet except by videoconference during the collaborative task and the two discussion phases. We note that while we had 5 baselines in the feasibility study, the time to explain them, set them up and focus the children added significant time to the experiment, and sitting still and staring fixedly was uncomfortable for the majority, and so we have reduced the number of baselines for the proposed study.

For the first discussion phase, the children were introduced over videoconference and it was suggested that they chat to get to know one another, and to discuss potential game strategies while the experimenters set up the next part of the experiment. The collaborative phase of the game was then played with the child peer partner, also for 7 min. During this phase the game board was presented on the left half of the screen and the videoconference featuring the other child was presented on the right half of the screen (see Figure 2A). The children's webcams were adjusted to capture their entire face and shoulders. A high-fidelity microphone captured the children's speech. Subsequently the children were instructed to chat freely while the experimenters set up the last part of the experiment. PsychoPy (Peirce et al., 2019) was responsible for maintaining the synchronization of the audio, video and fNIRS equipment.

## 9 What can we conclude from the feasibility study?

Five dyads of children participated, based on recruitment among local researchers and the social media accounts of a local babylab. The children in one dyad turned out to know one another beforehand, however they and the other four dyads were able to complete the task, and the data of all five are of high quality, and are included in the feasibility study analyses reported here. The five dyads are composed of 2 dyads of boys and 3 dyads of girls. The children had an average age of 10.2 years, with a standard deviation of 1.4 years.

**Figure 5:**
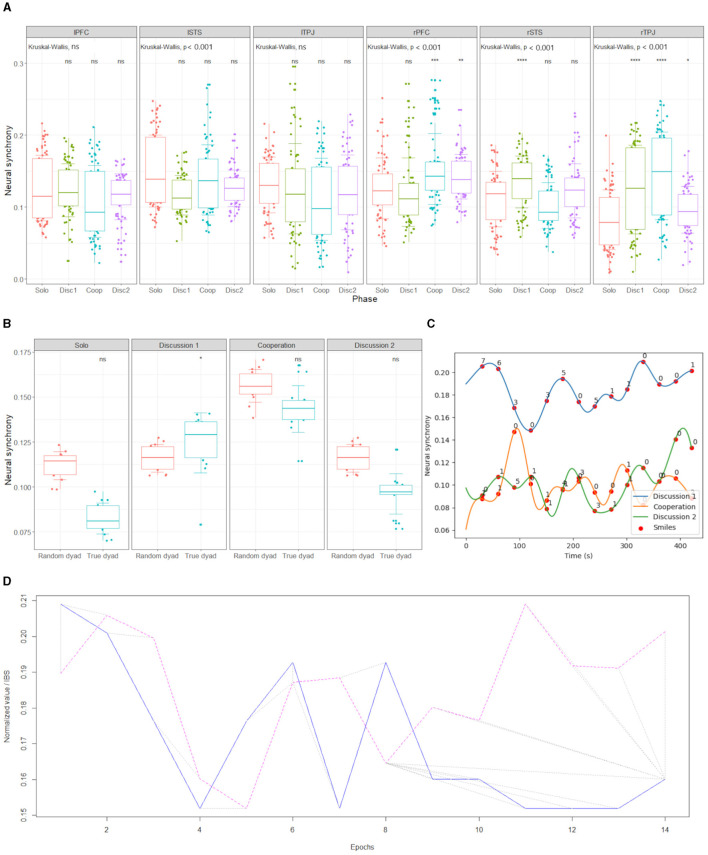
**(A)** IBS across different experimental phases and ROIs was investigated for the five dyads. The Kruskal-Wallis test, a non-parametric analysis, revealed significantly elevated IBS in the right hemisphere and the left STS. Additionally, when comparing to the solo phase as reference, the non-parametric *t-*test identified significantly higher IBS levels specifically in the right TPJ during the collaboration and both discussion phases. **(B)** Analysis of authentic vs. random dyads for the right TPJ for each experimental phase. **(C)** IBS evolution in the rTPJ during the social phases for a representative dyad. Red points represent the occurrence of smiles for the associated 30-second epoch. **(D)** Dynamic Time Warping (DTW) was carried out on the same dyad to assess pattern similarities between the occurrences of smiles (blue line) and IBS in the rTPJ (magenta line) during the initial discussion. The x-axis reflects normalized values, while the y-axis corresponds to epochs, with each epoch equivalent to 30 seconds of the initial discussion phase.

As described in the Supplementary material, analyses conducted on HbO values showed that IBS was significantly greater during the two interactive phases (collaboration and social) than during the solo phase, specifically in the right TPJ (Figure 5A). As evidence that the presence of IBS is due to social interaction and not to looking at the same stimulus materials, during the initial discussion period, IBS for the rTPJ was significantly higher for the authentic dyads than for the random pairing of children (Figure 5B). These results resemble those found in parent-child pairs, for example in Nguyen et al. (2020), as well as for adults in similar situations (Jiang et al., 2012). The fact that the STS did not demonstrate significantly higher IBS in the collaboration or discussion phases than in the solo phase, as has been found in a number of studies looking at perception of faces (e.g., Lee Masson and Isik, 2021), may result from the videoconference set-up where, for example, it is more difficult to assess whether the other person is meeting one's gaze. This suggests that data collected in a face-to-face condition with fewer children should also be included during the proposed study in order to ensure that data collected via videoconference is substantially similar to that collected face-to-face.

Analyses of conversational behaviors showed more smiles than other non-verbal behavior throughout the experiment, however the smiles were differentially distributed. For dyad 1 for example (two 10-year-old girls), while smiles were present throughout, smile frequency was higher during the two discussion phases than during the collaborative phase (see Figure 5C). Looking at the interaction between conversational behaviors and IBS, the smiles during the initial discussion phase coincided with heightened levels of IBS. Consequently, to investigate this association, a DTW analysis was conducted to assess the similarity in temporal dynamics between smile occurrences and IBS levels during this phase. This demonstrated evocative results, as the warping distance was small during the initial 8 epochs, that is, during the first 4 min (Figure 5D). In terms of paraverbal behaviors, laughter was found in all phases.

## 10 Limitations

The study proposed here is ambitious in its goals, but we believe that the results obtained justify the paradigm. Nevertheless, a number of limitations need to be addressed. The number of participants in the feasibility study is too small to draw conclusions about child-child interaction from these data. They do however indicate the feasibility of carrying out hyperscanning of child-child dyads, over videoconference (for more details about the preliminary findings, see the Supplementary material). Longitudinal studies sometimes lose participants for subsequent sessions. We mitigate this risk by collecting more data than recommended by the power analysis, by working with schools, rather than bringing participants into the lab, and by conducting subsequent sessions during a range of dates, rather than insisting on one single date. However, in cases where children drop out, we rely on the statistical methods described above to account for missing data and smaller samples. The study must be carried out by videoconference, both to allow the matching of unfamiliar children, and to allow commonality in screen presence between the child-child and child-VP studies. Among other reasons, this allows us to take into account in both studies the influence of blue light emitted by screens, which may cause an increase in saccadic eye movements (Lee Masson and Isik, 2021). However, we recognize that data collected by videoconference may demonstrate attenuated IBS, and issues with the localization of eye gaze. Both of these limitations are addressed above, but must be kept in mind. Finally, in this study, we have focused on spatial rather than temporal resolution, by using fNIRS, but some short-term phenomena may not be captured. To address this, a future study might couple fNIRS to EEG for improved temporal resolution.

## 11 Discussion

By integrating the four types of data described above, we aim to gain a deeper understanding of how pairs of children deploy conversational behaviors to establish bonds during middle childhood, a developmental stage characterized by a heightened focus on peer interaction. This integrated approach can also shed light on the neural processes involved in the development of interpersonal rapport over the course of a budding relationship, and the development of rapport-building over the course of middle childhood, as well as the role of rapport in educational performance. We also argue that a better understanding of these mechanisms can play a translational role in improving technologies for education, in particular the development of virtual peers, artificial intelligence partners in learning. To achieve the goals laid out above, we propose a novel multimodal approach using hyperscanning via functional near-infrared spectroscopy to study the neural, behavioral, psychological and performance correlates of rapport in middle childhood, during remote social and task exchanges in an ecologically-valid context of collaboration. While literature directly addressing our topic is limited, an extensive review of literature in social neuroscience and concerning children's multimodal conversational behavior, and interpersonal bond formation allows us to formulate guiding hypotheses. Additionally, a feasibility study demonstrates our ability to collect high-quality hyperscanning data from children engaged in videoconference collaboration and conversation, and to analyze its relationship to these other types of data.

The specific methodology proposed here is designed to address the paucity of literature concerning how children in middle childhood build social bonds with their peers, and the nature of those peer relationships. It is further motivated by the need to develop new kinds of educational tools, based on AI techniques, but that are optimized for the way children naturally learn, by including a social infrastructure for learning. We choose to pair children across videoconferencing, an increasingly researched communication modality (Bodur et al., 2023) that became commonplace even among young children during the pandemic. To achieve these goals, we proposed a fNIRS paradigm that includes an engaging and ecologically valid task, aiming to evoke naturalistic conversations between unfamiliar children. We also propose a novel analysis pipeline to examine the relationship among conversational behaviors, interpersonal rapport, educational performance, and IBS. We argue that this multimodal, multilevel, and multidisciplinary design offers a more well-rounded approach to studying the development of social bonds between peers during a crucial period of children's lives.

Other methods, drawn from machine learning, could improve the assessment of interpersonal coordination of conversational behavior, and its relationship with task performance, rapport, and neural synchrony. Multimodal learning with transformers is one place to look for these analytic tools, and their development is currently a major focus of attention, as is their application to important fields of inquiry such as clinical diagnostics (Zhou et al., 2023). Other methods, too, may provide a more informative analysis of the temporal dynamics of each of these behavior streams, and the relationships among them, such as the relationship between rapport and IBS over the course of the 6 weeks. TITARL (Guillame-Bert and Crowley, 2012) is one such option, that we have used before (Zhao et al., 2016; Madaio et al., 2017b) and that produces a series of rules to predict an outcome event (such as high rapport) based on a series of prior events (such as laughter followed by mutual gaze, but in the absence of praise). Such methods are particularly important for fNIRS studies where temporal resolution is on the level of seconds rather than milliseconds, and investigating task components is both possible and necessary.

The initial experiment will take place in France, with French-speaking children, but a subsequent study, not addressed in the current article, may add an American English-speaking sample, as social bond-building behavior differs in interesting ways between the two cultures, which are usually seen as quite similar (Béal, 2010). This first instance of the experiment will focus on neurotypical individuals and questions addressing neurotype will therefore be included in the parent questionnaire. We envision subsequent iterations of the study to include neurodivergent individuals, in line with our prior work with that population (Tartaro et al., 2014).

Nevertheless, the methodology and study proposed here do hold the promise of demonstrating the range of socio-cognitive factors that impact children's ability to build rapport with their peers, and the neural signature of that ability. We hope, as well, to better understand the brain networks implicated in these processes at each stage of middle childhood, as there is a dearth of ecologically-valid prior work on the neuroscience of social interaction among peers in this age group. Our feasibility study already provides tentative neural correlates of rapport among pairs of children. We hope future studies will validate those correlates but also uncover the neural mechanisms of rapport at both intra- and inter-personal levels.

## Ethics statement

The studies involving humans were approved by the Research Ethics Committee of the Paris Cité Université in May 2022, n°IRB: 00012022-31. The studies were conducted in accordance with the local legislation and institutional requirements. Written informed consent for participation in this study was provided by the participants' legal guardians/next of kin.

## Author contributions

JB: Conceptualization, Writing – original draft, Writing – review & editing, Investigation, Methodology, Visualization. GD: Writing – original draft, Formal analysis. JC: Conceptualization, Funding acquisition, Methodology, Project administration, Resources, Supervision, Writing – original draft, Writing – review & editing.
