# Supplementary material for: Bringing together multimodal and multilevel approaches to study the emergence of social bonds between children and improve social AI
Source: Front Neuroergon. 2024 May 17;5:1290256. doi: 10.3389/fnrgo.2024.1290256 (PMC11140154; doi:10.3389/fnrgo.2024.1290256)
Supplement: Supplementary file 1 [file Presentation_1.pdf]

Supplementary Materials

1. Data Security and Confidentiality

In order to respect children's right to their image, through the authorization of their legal representatives, a passage in the consent form is dedicated to this topic, specifying that the video recordings collected are stored and encrypted on a server to which only the lab's researchers with special authorization have access and that all of these persons have undergone ethical training for the use of this kind of data. In addition, the consent form gives legal guardians the right to choose the level of access to their child's image. The legal guardian can choose to allow no access to anybody other than the researchers, or for the images to be used for academic purposes (photos or short video clips shown in conferences), or for access to be given to people outside of the researchers' lab group for annotation purposes (e.g., Prolific or similar) in which case identifying information is removed, and where each annotator sees only a 30-second slice of video. By signing and initialling their choice for each part of the consent, the child's legal representative chooses what conditions to allow. It was under these conditions that the data for the pilot study were collected, and that the data for the proposed study will be collected.

2. Preliminary Results from Feasibility Study

Due to the limited sample size (n=5 dyads), it was inappropriate to run the kind of GLM analysis we will conduct for the proposed study. Consequently, Kruskal-Wallis comparisons were conducted, revealing strongly significant differences in IBS between phases for the three ROIs located in the right hemisphere and for the left STS (Fig. 5A). Additionally, utilizing the solo phase as the reference group, multiple pairwise Wilcoxon comparisons, Bonferroni corrected, revealed notably elevated levels of IBS in the right TPJ during all interactive phases (Solo-Discussion1: p-value = 1.01e-08 ****, Solo-Collaboration: p-value = 1.59e-08 ****, Solo-Discussion2: p-value = 3.72e-0.2 *). Based on these findings, we opted to center our subsequent analyses specifically on this particular ROI. We therefore generated multiple random pairs (n=200) by computing IBS between the target child's time-series paired with another child from a different dyad during the same phase. This process was repeated for each experimental phase, resulting in 14 averaged IBS values derived from these 200 simulated dyads for each phase. Furthermore, we calculated the mean IBS across the 5 authentic dyads and contrasted this with the averaged IBS of the 200 simulated dyads using a Wilcoxon test for each experimental phase (Fig. 5B). Our analysis revealed statistically significant higher levels of IBS for the authentic dyads compared to the false dyads, but only during the initial discussion phase and not during the collaboration nor the second discussion. In terms of nonverbal behavior, smiles were more frequent and lasted longer than any of the other nonverbal behaviors, and this was the case in all of the phases. This led us to prioritize smiles in our subsequent analyses of nonverbal behavior. The highest frequency of smiles was found in discussion one, where the children first met (see analysis of a representative dyad depicted in Fig. 5C). This is perhaps not surprising as, contrary perhaps to popular belief, smiles are more likely to co-occur with embarrassment than with happiness (Ambadar et al., 2009). To illustrate our proposed approach, we conducted a DTW analysis on the occurrence of smiles for both children in the representative dyad during the initial discussion, segmented into 30-second intervals, as described above, and the associated IBS in the rTPJ corresponding to these intervals. Our objective was to ascertain whether there was evidence of pattern similarity between these two curves. The results were encouraging, showing pattern similarity in the initial 8 epochs, equivalent to the first 4 minutes of the experimental phase. This similarity is reflected in small warping distances, with a slight temporal offset observed for the IBS (magenta line), as described in Chang et al. (2022).

It should be kept in mind that these are preliminary analyses from 5 dyads. While they may hold some interest, their primary purpose is to confirm the feasibility of a hyperscanning protocol with dyads of unfamiliar children in middle childhood, interacting with one another at a distance, and the analysis of data collected in this manner. We believe they have successfully fulfilled that function.

Ambadar, Z., Cohn, J. F., & Reed, L. I. (2009). All Smiles are Not Created Equal: Morphology and Timing of Smiles Perceived as Amused, Polite, and Embarrassed/Nervous. *Journal of Nonverbal Behavior*, *33*(1), 17–34. https://doi.org/10.1007/s10919-008-0059-5

Chang, C. H. C., Nastase, S. A., & Hasson, U. (2022). Information flow across the cortical timescale hierarchy during narrative construction. *Proceedings of the National Academy of Sciences*, *119*(51), e2209307119. https://doi.org/10.1073/pnas.2209307119
